# Leucine-rich repeat proteins of *Leptospira interrogans* that interact to host glycosaminoglycans and integrins

**DOI:** 10.3389/fmicb.2024.1497712

**Published:** 2024-11-26

**Authors:** Bruno B. Foltran, Aline F. Teixeira, Eliete C. Romero, Luis G. V. Fernandes, Ana L. T. O. Nascimento

**Affiliations:** ^1^Laboratório de Desenvolvimento de Vacinas, Instituto Butantan, São Paulo, Brazil; ^2^Programa de Pós-Graduação Interunidades em Biotecnologia, Instituto de Ciências Biomédicas, Universidade de São Paulo, São Paulo, Brazil; ^3^Centro de Bacteriologia, Instituto Adolfo Lutz, São Paulo, Brazil; ^4^Infectious Bacterial Disease Research Unit, U.S. Department of Agriculture (USDA) Agricultural Research Service, National Animal Disease Center, Ames, IA, United States

**Keywords:** *Leptospira*, leptospirosis, LRR-proteins, adhesion, pathogenesis

## Abstract

Pathogenic spirochaetes of the genus *Leptospira* are the etiological agents of leptospirosis, a zoonotic infection worldwide. The disease is considered an emerging and re-emerging threat due to global warming, followed by heavy rainfall and flooding when outbreaks of leptospirosis occur. Adhesion to host tissues is mediated by surface/extracellular proteins expressed by pathogens during infection. Leucine-rich repeat (LRR) domain-containing proteins seem to be important for the virulence of pathogenic *Leptospira* and their role has been recently examined. Here, we report the characterization of two LRR-proteins encoded by LIC11051 and LIC11505. They present 7 and 17 LRR motifs, respectively. LIC11051 was found mainly in the P1 subclade, whereas LIC11505 was identified with higher identity in subclade P1, but was also found in subclades P2, S1, and S2. The recombinant proteins were recognized by antibodies in leptospirosis serum samples, suggesting their expression during infection. rLIC11505 contains a broad spectrum of ligands, including GAG and integrin receptors, whereas rLIC11051 showed limited binding activity. The attachment of proteins to ligands was specific, dose-dependent, and saturable. Compared to their role in adhesion, both proteins were shown to be secreted, with the ability to reassociate with the bacteria. Taken together, our data suggested that LIC11051 and LIC11505 participate in leptospiral pathogenesis. To the best of our knowledge, this is the first report showing leptospiral LRR-proteins exhibiting GAG and integrin receptor-binding properties.

## Introduction

Leptospirosis is a neglected tropical disease of human and veterinary concern worldwide. The disease is responsible for more than a million human cases per year, including ~58,000 deaths (Costa et al., 2015). Symptoms of leptospirosis may vary from asymptomatic self-limiting to mild flu-like disease, which can progress to more severe manifestations characterized by multi-organ failure, jaundice, and hemorrhage, called Weil's disease (Faine, 1994). Leptospirosis-associated pulmonary hemorrhage syndrome (LPHS) is an even more life-threatening form of the disease with a mortality rate of over 50% (Marotto et al., 1999).

In urban areas, asymptomatic *Rattus novergicus* is the main reservoir host, shedding bacteria through their urine, contaminating water, and soil. Transmission occurs directly from contact with infected animal fluids or indirectly through contact with a contaminated environment (Adler and de la Peña Moctezuma, 2010; Bharti et al., 2003), by active penetration of leptospires through abraded or sodden skin and mucosa.

Some pathogenic bacteria can escape the host's innate immune barriers, spread across the bloodstream, and finally reach target organs such as the lungs, liver, and kidneys. A critical feature of the infection process used by pathogens, including *Leptospira*, is adhesion to host cells through interactions with cell adhesion receptors and extracellular matrix (ECM) components (Ito and Yanagawa, 1987).

Due to their location, bacterial surface proteins and exoproteins may play a critical role in pathogenesis by interacting with the host ECM, plasma components, and cell receptors (Daroz et al., 2021; Eshghi et al., 2015; Evangelista et al., 2014). Many sequences predicted to encode surface-exposed and/or secreted proteins with either conserved domains or unknown functions have been identified in the genome of *L. interrogans* serovar Copenhageni (Nascimento et al., 2004a). Several recombinant proteins have been shown to interact with a myriad of host components, including laminin, fibronectin, plasminogen (PLG), fibrinogen, and components of the complement system (Oliveira et al., 2011). The adhesion of *L. interrogans* and recombinant proteins to several cell lines cultured *in vitro*, such as endothelial cells, fibroblasts, and kidney epithelial cells, has also been reported (Evangelista et al., 2014; Robbins et al., 2015; Takahashi et al., 2022).

LRR-proteins belong to an important family of proteins, characterized by the presence of regions containing various leucine residues (Bella et al., 2008), and have recently been investigated in *Leptospira* spp. (Miras et al., 2015). An important contrasting feature of leptospiral LRR-proteins protein-coding sequences is their presence in pathogenic and saprophyte genomes. For instance, *L. interrogans* and *L. borgpetersenii* encode at least 20 and five LRR-containing proteins, respectively, in contrast to the saprophyte *L. biflexa* genome, which possesses only one annotated LRR-protein-encoding gene (Picardeau et al., 2008; Picardeau, 2017; Eshghi et al., 2019). The predominance of LRR-proteins in pathogenic species of *Leptospira* suggests that these proteins may actively contribute to leptospiral infections, making them interesting targets for research. To date, some LRR-proteins of pathogenic *Leptospira* spp. have been characterized (Miras et al., 2015; Hsu et al., 2020; Hsu and Yang, 2022; Prapong et al., 2023), and the recombinant protein rLIC10831 from *L. interrogans* was shown to bind VE- and E-cadherins (Eshghi et al., 2019), whereas rLIC11098 exhibited a larger spectrum of interaction with host molecules (Gaspar et al., 2024).

In the present study, we characterized two novel recombinant LRR-proteins, encoded by the genes LIC11051 and LIC11505, identified in the genome sequences of *L. interrogans* serovar Copenhageni (Nascimento et al., 2004a,b) and in the global proteome of the same strain (Malmström et al., 2009). We showed that one of the recombinant proteins, rLIC11505, presents a broad spectrum of ligands, including GAGs and integrin receptors, while rLIC11051 exhibits a restricted binding profile. Our data suggests that these proteins participate in leptospiral pathogenesis.

## Materials and methods

### Macromolecules and antibodies

The macromolecules collagen IV (human placenta, C7521), collagen I (rat tail, C3867, and calf skin, C8919), elastin (human aorta, F5881), E-cadherin (human recombinant, 5085), cellular fibronectin (human foreskin fibroblasts, 2518), laminin (human placenta, L6274), plasminogen (human plasma, P7999), plasma fibronectin (human plasma, F2006), vitronectin (human plasma, V8379), fibrinogen (human plasma, F4883) chondroitin sulfate (shark cartilage, C4384), chondroitin sulfate B (porcine intestinal mucosa, C3788) chondroitin 4 sulfate (bovine trachea, 27042), heparin (porcine intestinal mucosa, H4784), heparan sulfate (bovine kidney, H7640), fetuin (fetal serum bovine, F3385), and BSA (bovine serum albumin, A7906) were purchased from Sigma-Aldrich, USA. Factor H (human, A137), C3b (human, A114), C4b (human, A108), C4bp (human, A109), C7 (human, A124), C8 (human, A125), and C9 (human, A126) were purchased from Complement Technology (USA). Integrins α8, α5β1, αVβ1, αLβ2, αVβ3, αVβ5, and αVβ6 were purchased from Santa Cruz Biotechnology (CA, USA). The commercial antibodies used were peroxidase (HRP)-conjugated anti-mouse IgG (A9044), mouse anti-PLG antibody (WH0005340M1), and mouse monoclonal anti-polyhistidine-peroxidase antibody (A7058) acquired from Sigma-Aldrich, USA.

### *Leptospira* strains and culture

*Leptospira* strains utilized in this work comprise pathogenic *L. interrogans* serovar Copenhageni strain FIOCRUZ L1-130 (recently isolated from an animal, <5 *in vitro* passage) and *L. interrogans* serovar Copenhageni strain M20 (high-passage, culture-attenuated strain), and the saprophytic strain *L. biflexa* serovar Patoc, strain Patoc1. Cells were grown at 28°C under aerobic conditions in liquid EMJH medium (Difco, BD, Franklin Lakes, NJ, USA) supplemented with 10% (v/v) *Leptospira* enrichment EMJH (BD, Difco).

### Bioinformatics analysis

The genes LIC11051 and LIC11505 were identified on chromosome I of *L. interrogans* serovar Copenhageni strain Fiocruz L1-130 and subjected to *in silico* analysis to predict the conformation of encoded proteins, the presence of conserved domains, and cellular location. WebCutter (https://heimanlab.com/cut2.html) was used to predict restriction enzyme sites within the nucleotide sequences. The SMART program (Letunic et al., 2021; https://smart.embl-heidelberg.de/) was used to identify protein-conserved domains. To predict the signal peptide and N-terminal lipidation, SignalP (Teufel et al., 2022; https://services.healthtech.dtu.dk/services/SignalP-6.0/), and LipoP (https://services.healthtech.dtu.dk/services/LipoP-1.0/). Cellular localization prediction was also performed with the CELLO (Yu et al., 2006; http://cello.life.nctu.edu.tw/) and PSORTb webserver (Yu et al., 2010; https://www.psort.org/psortb/) For the 3D structural analysis, amino acid sequences of each protein were submitted to the online tool AlfaFold (Protein Structure Database, https://alphafold.ebi.ac.uk/) and the highest score alignments were selected.

The similarity of the LIC11051 and LIC11505 amino acid sequences of *L. interrogans* serovar Copenhageni strain Fiocruz L1-130 compared to 64 other *Leptospira* species classified by Vincent et al. (2019) was performed using the BLAST web server (Altschup et al., 1990; https://blast.ncbi.nlm.nih.gov/Blast.cgi). Coverage and identity percentages were used to calculate the conservation value (*c*-value) of the LRR protein orthologs. The c-value was expressed as an index between 0.0 (not present) and 1.0 (conserved; Lopes et al., 2019; Nascimento Filho et al., 2024) and was calculated as follows:


$$\begin{array}{l} c\;value=\frac{identical+similar\;amino\;acids}{2}\;.\;coverage \end{array}$$

Heatmap images and data analysis were performed using Seaborn version 0.13.2 (Waskom, 2021), NumPy version 1.24.0 (Harris et al., 2020), Pandas version 2.2.2 (Reback, 2020), and Matplotlib version 3.9.0 (Hunter, 2007) at the Jupyter notebooks that are hosted by Colab (https://colab.research.google.com).

### Cloning, expression and purification of recombinant proteins

The genomic DNA of *L. interrogans* serovar Copenhageni was used as a template to obtain target genes by PCR. Two pairs of oligonucleotides were designed as follows: forward, 5′-ATCGGGATCCCACTACCTAAGCTCCAAG-3′ (*BamH*I, underlined) and reverse−5′-ATCGGGTACCTCAAGAAAGATAAAGTACTAC-3′ (*Kpn*I, underlined) for LIC11051; and forward−5′-ATCGCTCGAGGACGAAGTAAAACC-3′ (*Xho*I, underlined) and reverse−5′-ATCGCCATGGCTAATAAACTTCAAAAATTAATTTTAC-3′ (*Nco*I, underlined) for LIC11505, excluding the nucleotides coding for the predicted signal peptide when present. Restriction sites were added to the 5′ region of the oligonucleotides for cloning into the pAE vector, which allowed the expression of recombinant proteins containing six histidine residues in the N-terminal region (Ramos et al., 2004). After amplification, the fragments were purified using the GFX™ PCR DNA and Gel Band Purification kit (GE Healthcare, Chicago, Illinois, USA), digested with the appropriate restriction enzymes according to the manufacturer's instructions, and ligated *in frame* into the pAE vector previously digested with the same enzymes by T4 DNA ligase. Successful cloning was verified by DNA sequencing using the chain termination method (Sanger et al., 1977) on an ABI automatic sequencer (PE Applied Biosystems, Foster City, CA, USA).

Recombinant plasmids were used to transform the expression strain *E. coli* Star™ (DE3) pLysS. Cells were grown in 200 mL of medium inoculated with 1% of an overnight saturated culture supplemented with ampicillin (100 μg/mL) and chloramphenicol (34 μg/mL). The cultures were incubated at 37°C with shaking until they reached an optical density (OD 600 nm) of 0.6, and then protein expression was induced by adding 0.1 mM IPTG (isopropyl β-d-1-thiogalactopyranoside) and incubating at 18°C for 16 h with shaking. Cultures were centrifuged at 6,000 × *g* for 10 min at 4°C, resuspended in 20 mL lysis buffer [20 mM Tris-HCl [pH 8.0 for LIC11051; pH 6.8 for LIC11505], 200 mM NaCl, 200 μg/mL lysozyme, 2 mM phenylmethylsulfonyl fluoride [PMSF], and 1% Triton X-100], and sonicated for 10 min on ice to disrupt the cells. After lysis, the mixture was centrifuged again at 10,000 × *g* for 10 min at 4°C, and the supernatant containing soluble proteins was purified using metal affinity chromatography (Ni^2+^). Contaminant proteins were removed using imidazole gradients (50 mL of each imidazole concentration was used for the washing steps: 20 and 40 mM for both proteins, with an additional 60 mM wash for LIC11505). The proteins were then eluted with 20 mL of imidazole (60 mM for LIC11051 and 500 mM for LIC11505), followed by three dialysis steps of 1 h each in Tris-NaCl buffer [20 mM Tris-HCl [pH 8.0 for LIC11051; pH 6.8 for LIC11505], 200 mM NaCl] to remove imidazole.

### Analysis of the secondary structure of recombinants by circular dichroism

The recombinant proteins were dialyzed in 1 liter of sodium-phosphate buffer (0.5 M Na_2_HPO_4_; 1 M NaH_2_PO_4_; pH 7.4) at 4°C, three times at 1-h intervals. CD was measured at 25°C using a cuvette with a 1 mm optical path at intervals of 0.5 nm/s and captured on a Jasco J-810 model spectropolarimeter (Japan Spectroscopic, Japan) equipped with a Peltier unit for temperature control. The expressed spectra were measured in terms of residual molar ellipticity (Θ × L × C × 10 ^3^), where Θ (degrees) is the ellipticity, L (cm ^2^) refers to the optical path length, and C (dmol −1) is the concentration of the protein. The average readings were used for the analysis of secondary structures by submitting them to BeStSel software (https://bestsel.elte.hu/index.php).

### Polyclonal antisera production and antibody titration

BALB/c mice (18–22 g), five animals/cage, were immunized subcutaneously with 10 μg of each recombinant protein adsorbed in 12.5% Al (OH)_3_ with two boosters at intervals of ~15 days. No criteria were set for including or excluding animals. The animals were bled via the retro-orbital plexus before immunization as a non-immune control, and after each immunization. There were no exclusions. The collected blood was stored at 37°C for 30 min, and the clot was centrifuged at 2,500 × *g* at 4 for 10 min. After centrifugation, the collected serum was stored at −20°C. Next, the sera were titrated by ELISA, in which 250 ng of each recombinant protein was immobilized for 16 h at room temperature per well, and subsequently blocked with a PBS-0.05% Tween 20 (PBS-T) solution containing 10% skimmed milk. Then, animal sera at different serial dilutions were added to the wells and incubated for 1 h, followed by washing and incubation with an anti-mouse IgG secondary antibody conjugated to peroxidase. Reactivity was detected by adding 1 mg/mL OPD (o-phenylenediamine) with 1 μL/mL H_2_O_2_, after 10 min, the reaction was stopped by adding 50 μL of 2 M H_2_SO_4_ and the absorbance (OD 492 nm) was measured using a Multiskan-FC microplate reader (Thermo Fisher Scientific, Helsinki, Finland). The antibody titer was defined as the inverse of the last dilution that resulted in an absorbance above the threshold value of 0.1 (492 nm). Blank controls without serum were used in all experiments, and three independent experiments were performed.

### Detection of target proteins in *Leptospira* spp. and evaluation of antibody cross-reactivity

Mouse polyclonal sera were used to detect the respective native proteins in the total cell lysate samples of *L. interrogans* serovar Copenhageni Fiocruz L1-130 (pathogenic and virulent, <5 *in vitro* passages), *L. interrogans* serovar Copenhageni strain M20 (pathogenic, attenuated in culture), and *L. biflexa* serovar Patoc strain Patoc1 (saprophyte). Native proteins were detected in two additional fractions: secreted and SDS-soluble bacterial membrane proteins. For whole cell lysates, cultures were grown in 10 mL, cells were centrifuged at 6,000 × *g* for 15 min at room temperature, and the sediment was washed with PBS. The cell suspension was centrifuged again, and the recovered cells were resuspended in 150 μL of PBS. Optical density (OD) values were measured, and lysates were prepared. For secreted proteins, 100 mL of virulent *L. interrogans* serovar Copenhageni strain Fiocruz L1-130 culture was centrifuged, the medium discarded, and the pellet washed with 10 mL of PBS. Next, cells were resuspended in 10 mL PBS and incubated at 30°C overnight for protein secretion. Leptospiral cells were centrifuged, and the supernatant was recovered and concentrated 10-fold using Amicon ultrafiltration with a molecular weight cut-off of 10 kDa (Millipore) and dialyzed into 10 mM ammonium bicarbonate. To isolate SDS-soluble membrane proteins, cell lysis was performed followed by three successive rounds of sonication for 30 s at 20 kHz. Whole cells were removed by low-speed centrifugation (3.000 x *g*, 15 min), and the supernatant collected was subjected to ultracentrifugation at 27,000 × g for 20 min at 4°C. The pellet containing the proteins was resuspended in PBS containing 2% SDS and stirred overnight at 37°C. The SDS-soluble fractions were collected after centrifugation at 17,000 × *g* for 20 min at room temperature. SDS extraction was repeated once for 4 h at 37°C. The SDS present in the samples was removed using Extract gel columns and the SDS-Out SDS precipitation kit (Thermo Scientific; Teixeira et al., 2022). Native proteins were detected by immunoblotting. To demonstrate the cross-reactivity of the antibodies, an ELISA was performed, in which 1 μg of recombinant protein was coated onto a 96-well plate at 4°C overnight and subsequently blocked with a PBS-T solution containing 10% skimmed milk. Polyclonal anti-rLIC11051 was allowed to interact with immobilized rLIC11051 and rLIC10505, and conversely, polyclonal anti-rLIC10505 was incubated with both recombinant proteins. Pre-immune serum was used as negative control. After a 1 h incubation step, the plates were washed and incubated with a secondary anti-mouse IgG antibody conjugated with peroxidase, and reactivity was detected as previously described.

### SDS-PAGE and immunoblotting

Samples were mixed with denaturing buffer (10% SDS, 50% glycerol, 0.5% bromophenol blue, and 3% β-mercaptoethanol) and heated at 96°C for 10 min. Proteins were separated by sodium (sulfate-polyacrylamide gel electrophoresis) on 12% polyacrylamide gels. For protein visualization, the gels were stained with Coomassie Brilliant Blue (Sigma). For immunoblotting, proteins were electrotransferred onto nitrocellulose membranes (Hybond ECL; GE Healthcare, Chicago, IL, USA) using semidry transfer equipment. Membranes were blocked with PBS-0.05% Tween 20 (PBS-T) containing 10% skimmed milk for 2 h at 37°C, followed by incubation with anti-rLIC antibodies raised in mice (1:10.000), for 2 h at 37°C. Then, the membranes were washed three times for 5 min each and incubated for 1 h with HRP-conjugated anti-mouse IgG, diluted 1:5000 (Sigma) in PBS-T with 1% milk, followed by three 5-min washes. Protein reactivity was determined using an enhanced chemiluminescence (ECL) western blotting analysis system (GE Healthcare).

### Microscopic agglutination test and protein reactivity with human serum samples

MAT was performed as previously described by Faine et al. (1999). Paired human serum samples at the onset (MAT-negative) and in the convalescence phase (MAT-positive) from leptospirosis-confirmed samples were obtained from the serum collection of the Instituto Adolfo Lutz, São Paulo, Brazil. The antibody titer increases from MAT- to MAT+ and MAT is considered positive above titer 200. The reactivity of the recombinant proteins was investigated against 16 paired human leptospirosis serum samples at the onset (MAT-negative) and convalescent phases (MAT-positive). For this, 250 ng of each recombinant protein in carbonate buffer (0.1 M pH 9.6) was immobilized in 96-well ELISA plates and incubated overnight. The plates were washed three times with PBS-T and blocked with 200 μL of PBS-T containing 10% skimmed milk. Next, serum samples were added in duplicate to the wells at 1:100 dilution and incubated for 2 h at 37°C. Reactivity was assessed by incubation with HRP-conjugated anti-human IgG (1:5,000), followed by incubation with the OPD substrate, as described above. The cutoff values, above which a sample is considered positive, were defined as the mean plus three standard deviations of the absorbance values of seven serum samples from NHS.

Three independent experiments were performed.

### Binding of recombinant proteins to *L. interrogans* surface

High-passage *L. interrogans* Fiocruz L1-130 cells were centrifuged (3,000 × *g*, 10 min) at room temperature. The pellet was resuspended in 10 mL of sterile PBS, centrifuged again, and resuspended in PBS at a concentration of 10^9^ cells/ml. In the ELISA plate, the wells were coated with 10^8^ cells by incubation at room temperature for 16 h. BSA was also used for coating the wells, as a negative control. Wells were then blocked with a blocking solution (PBS containing 10% skimmed milk) for 2 h at 29°C. Serial concentrations of each recombinant protein were added to the wells and allowed to interact for 1 h at 29°C. After washing with PBS, anti-histidine antibody (1:5.000 in blocking buffer) was added, and detection was performed as previously described. Three independent experiments were performed.

### Interaction of recombinant proteins with host components

Individual components of the extracellular matrix (ECM), plasma, glycosaminoglycans (GAG), components of the complement system, and integrins were used to coat ELISA plate wells as follows: 1 μg/well of the host components (ECM, plasma, and complements), except for vitronectin (250 ng/well), GAGs, and integrins (100 μg/well). BSA and fetuin proteins were used as controls in all the experiments. The plates were incubated overnight at 4°C in PBS at a final volume of 100 μL/well and washed three times with PBS-T. A blocking solution containing PBS-T with 10% skimmed milk was then added and incubated for 2 h at 37°C in a final volume of 200 μL/well. After blocking, 1 μg/well of recombinant protein was added to a final volume of 100 μL of PBS-T with 1% skim milk and incubated for 2 h at 37°C. For the analysis of GAGs, two additional steps were performed: fixation with 100 μL/well of 2% paraformaldehyde in PBS for 30 min, followed by incubation with 2% glycine for 30 min at room temperature. After washing, mouse polyclonal antibodies against each recombinant protein were added and incubated for 1 h at 37°C (1:10,000 for LIC11051 and 1:20,000 for LIC11505 in blocking buffer). Detection of bound proteins was performed using HRP-conjugated anti-mouse antibody (1:5,000), followed by incubation with OPD substrate, as described above. Three independent experiments were performed.

### Dose response of recombinant proteins to host components

The host components that displayed significant interactions with the recombinant proteins were coated onto 96-well ELISA plates, as described above. After blocking, recombinant proteins at increasing concentrations were added to the plates and incubated for 2 h at 37°C. For the GAG dose-response assay, two additional steps were performed: paraformaldehyde fixation and incubation with glycine, followed by the same binding procedure. Incubation with the mouse antibody and detection of the reaction was performed as previously described. Dose-response curves and dissociation constant calculations were fitted using the “Non-linear Regression” tool of the GraphPad Prism v software.7, “Non-linear regression,” considering saturation binding with “one site-specific binding.” The statistical difference was analyzed using the *T*-test, comparing protein binding with the fetuin control. Three independent experiments were performed.

### Competition for PLG binding by aminocaproic acid

ELISA plate wells were coated with 1 μg of PLG for 16 h at 4°C. Then, the plate was washed with PBS-T, and a blocking solution was added, incubated for 2 h at 37°C. The recombinant proteins were then added together with two concentrations (2 and 20 mM) of the lysine analog aminocaproic acid (Sigma-Aldrich) for 2 h at 37°C. After washing with PBS-T, the reaction was detected, as previously described. Two independent experiments were performed.

### PLG capture from normal human serum

ELISA plates were coated with recombinant proteins or fetuin (1 μg/well) for 16 h at 4°C. After washing with PBS-T, a blocking solution was added for 2 h at 37°C. Normal human serum (Sigma-Aldrich) was added to each well at concentrations ranging from 0 to 30%. PLG binding to recombinant proteins was detected using a mouse anti-PLG monoclonal antibody (1:5,000) for 1 h at 37°C, followed by incubation with an HRP-conjugated anti-mouse antibody (1:5,000). The reaction was performed as described previously. Two independent experiments were performed.

### Plasmin formation assay

ELISA plates were coated with each recombinant protein or fetuin as previously described. After the washing and blocking steps, PLG was added to the wells (1 μg/well) and incubated for 2 h at 37°C. Plates were washed, and 5 ng of human urokinase PLG activator (uPA; Sigma-Aldrich) and 0.8 mM of PLA-specific substrate, D-valyl leucyl-lysine-p-nitroanilide dihydrochloride (Sigma-Aldrich) were added. Controls were prepared by omitting one of the reaction components (PLG, uPA, or the substrate). The reaction was carried out for 16 h at 37°C, and PLA activity was indirectly measured by the chromogenic substrate degradation, which generates an absorbance at 405 nm, measured using a microplate reader. Two independent experiments were performed.

### Statistical analysis

GraphPad Prism 6.05 software was used for statistical analysis (GraphPad Prism, USA).

For all experiments, a two-tailed *t*-test was used for data analysis. For dose-response analyses, curves were fitted using a “non-linear regression” analysis, taking into account reaction saturation with the “one site-specific binding” option. Values below the detection limit of the equipment (solution with absorbance around 0.05) were not considered reliable for statistical analysis and were therefore classified as non-reactive. One representative experiment is shown, selected from either two or three independent experiments performed.

### Ethics statement

This study was performed according to the guidelines outlined by the Brazilian National Council for Control of Animal Experimentation (CONCEA), which follows international guidelines for animal welfare and the principles of the 3Rs. Experimental protocols comply with the ARRIVE guidelines and were approved by the Ethic Committee on Animal Use of the Butantan Institute, São Paulo, Brazil (protocol no. 4425161222). Mice were housed in a BSL1 animal facility, in micro isolators with individual ventilation and temperature and light cycle control. Animals received food and water *ad libitum* and manipulation was performed by trained personnel. Human serum samples were obtained from the Adolfo Lutz Institute, São Paulo, Brazil, approved by the Ethics Committee (protocol no. 2.834.589). The National Research Ethics Commission (CONEP) approval is registered in “Plataforma Brasil site” (protocol CAAE 78770117.0.0000.8098).

## Results

### *In silico* analysis of LIC11051 and LIC11505

**Figure 1:**
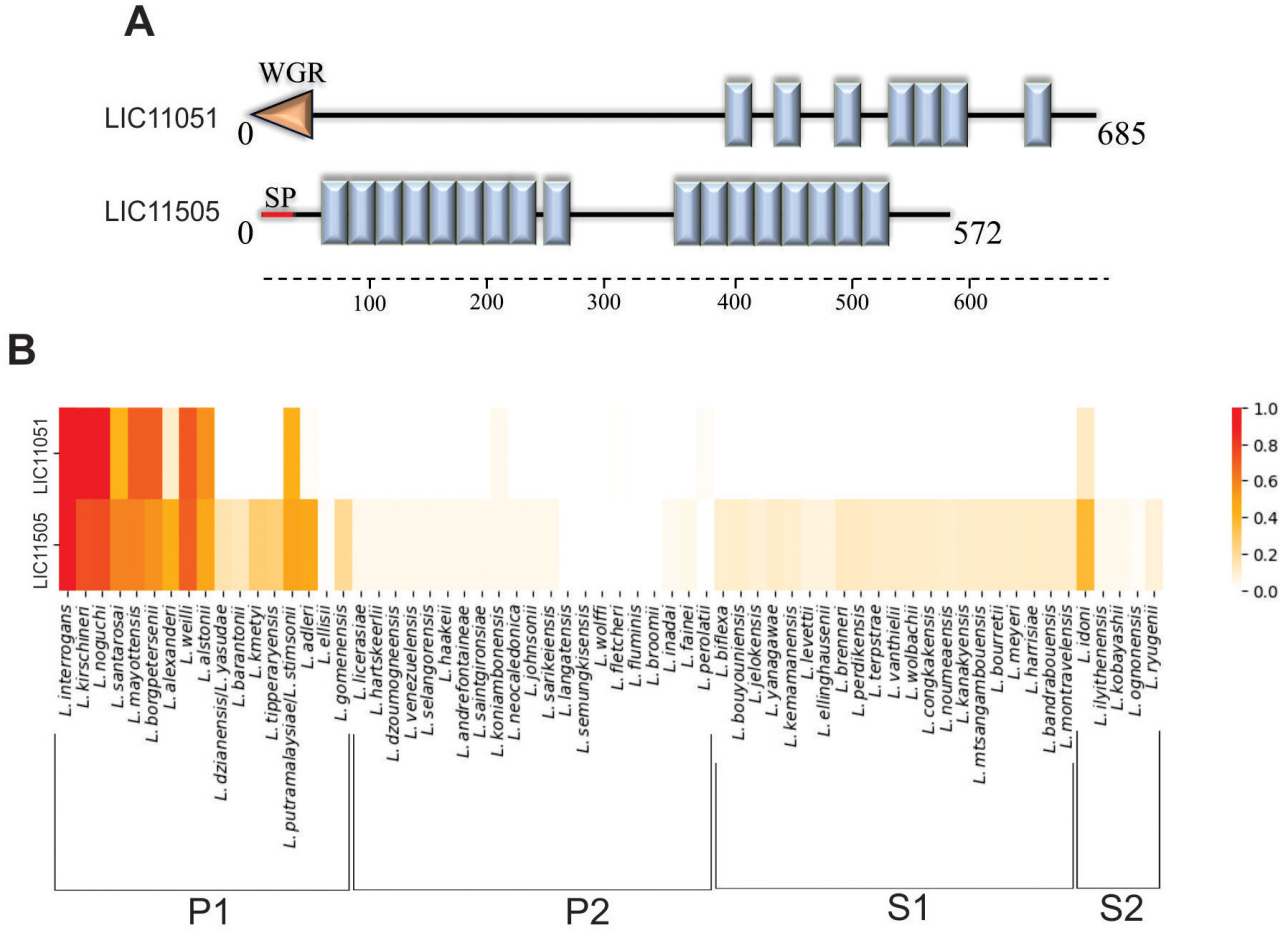
*In silico* analysis of LIC11051 and LIC11505 coding sequences. **(A)** Schematic representation of the LRR domains (blue boxes) in the sequence of LIC11051 and LIC11505 predicted by SMART. The orange arrow refers to the WGR domain and the red line refers to the signal peptide I (SPI). In **(B)** heatmap representation to visualize the conservation level of the coding sequences LIC11051 and LIC11505 of *Leptospira interrogans* serovar Copenhageni strain Fiocruz L1-130 among other species of *Leptospira*. The result was calculated based on the *c*-value where 1.0 means 100% conservation and 0.0 means no conservation.

The genes LIC11051 and LIC11505 were identified in *L. interrogans* serovar Copenhageni genome sequence (Nascimento et al., 2004b), and both encode proteins with LRR domains, known for their characteristic horseshoe fold of the α/β solenoid (Bella et al., 2008; Kobe and Kajava, 2001). The LRR domain is widely distributed and is found in several organisms, such as viruses, bacteria, archaea, and even eukaryotes (Helft et al., 2011). Conserved domain analysis of these proteins revealed the presence of seven LRR and one WGR (tryptophan, glycine, and arginine) domains for LIC11051 and 17 LRR domains for LIC11505 (Figure 1A). The amino acid sequences for LIC11051 and LIC11505 were also used to predict the presence of the signal peptide. No signal peptide was found for LIC11051, whereas LIC11505 displayed a signal peptide between amino acids 29 and 30, with a Signal Peptidase I (SpI) cleavage site (Juncker et al., 2003). Furthermore, cellular localization was predicted, with both proteins predicted to be extracellular by PSORTb (score: 9.45 for both proteins) and outer membrane proteins by CELLO (score: 2.147 for LIC11051 and 1.226 for LIC11505).

The 64 species of *Leptospira* were used to determine the conservation of the selected LRR-proteins among the pathogenic (subclade P1), intermediate (subclade P2), and saprophytic (subclades S1 and S2) groups of *Leptospira*. Both proteins were conserved among the subclade P1, exhibiting a higher c-value when compared to strains from P2, S1, and S2, which presented a low or 0 *c*-value (Figure 1B).

### Cloning and recombinant protein expression

**Figure 2:**
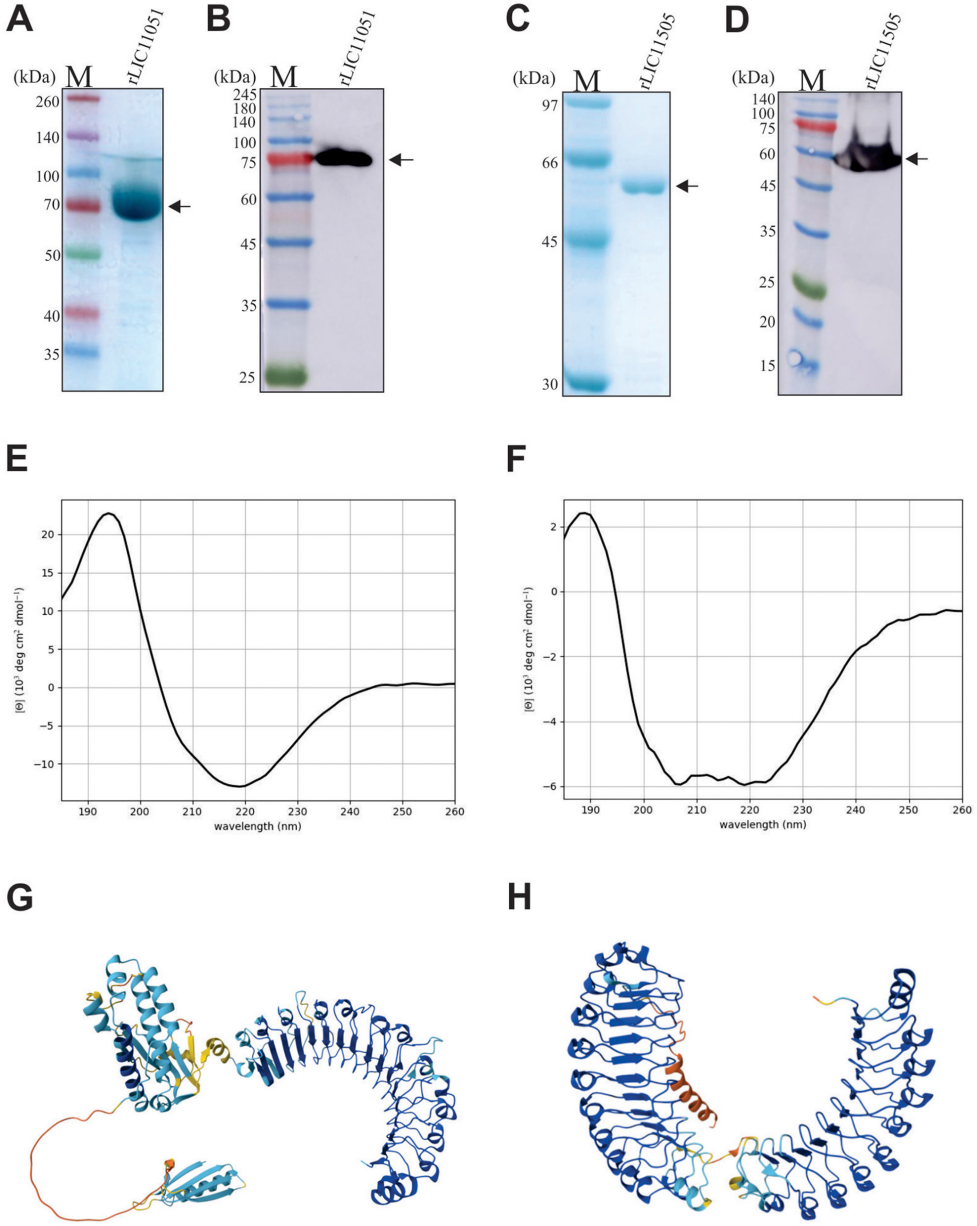
Recombinant protein expression, purification and secondary structure analysis of rLIC11051 and rLIC11505. **(A, B)** SDS-PAGE and Western blotting of rLIC11051 protein after dialysis, respectively; **(C, D)** SDS-PAGE and Western blotting using monoclonal anti-his antibodies of rLIC11505 protein after dialysis, respectively. Representative circular dichroism spectra of rLIC11051 **(E)** and rLIC11505 **(F)** proteins. 3D structure predicted by the AlphaFold database for LIC11051 **(G)** and LIC11505 **(H)**.

The LIC11051 and LIC11505 genes were amplified by PCR, with expected sizes of 2,076 and 1,656 bp, respectively, and were successfully cloned into the pAE expression vector (Ramos et al., 2004). Recombinant protein expression analysis showed that both rLIC11051 and rLIC11505 were present in the soluble fraction after bacterial lysis (data not shown). Protein purification was carried out using a metal affinity chromatography column, and the fractions of rLIC11051 and rLIC11505 were evaluated by SDS-PAGE. As observed by SDS-PAGE and western blotting with monoclonal anti-his antibodies, both recombinant proteins were successfully purified and dialyzed (Figures 2A–D), resulting in major bands at the expected molecular weight of 75 and 60 kDa and yields of 0.43 and 0.38 mg/mL, respectively. The secondary structure of the purified proteins was modeled using BeStSel software using experimental CD spectra. Graph-fitted curves are shown in Figures 2E, F. Structural analysis of rLIC11051 revealed 19.20% α-helix, 36.7% β-sheet, and 44.0% other structures, whereas rLIC11505 revealed 6.9% α-helix, 46.2% β-sheet, and 46.9% of other structures. The theoretical values for rLIC11051 were 31.4% for α-helix, 11.4% for β-sheet, and 57.2% for other structures. For rLIC11505, 38.6% α-helix, 11.0% β-sheet, and 50.4% of the other structures were found. The differences between the *in silico* and experimental results may be due to the conditions used in the purification and experimental tests of the proteins, such as temperature, pH, and buffer used. The tertiary structure of the recombinant proteins modeled by the AlphaFold database showed a structural arrangement compatible with that described previously (Bella et al., 2008; Kobe and Kajava, 2001; Miras et al., 2015). The results showed the presence of horseshoe folds (Figures 2G, H).

### Reactivity with leptospirosis serum samples

**Figure 3:**
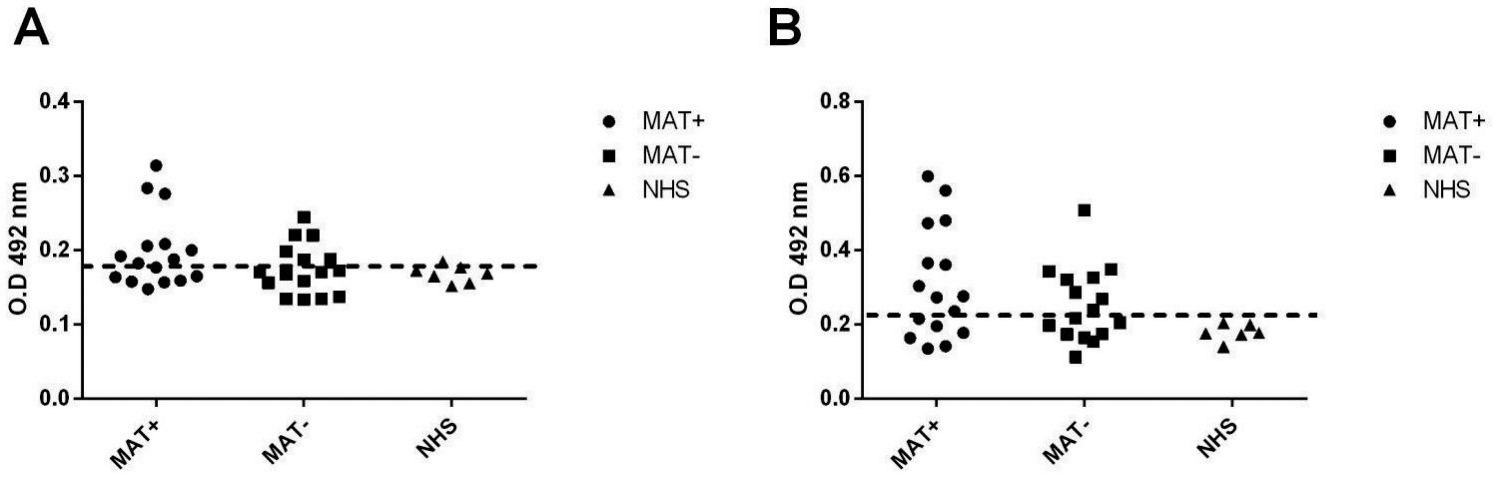
Reactivity of recombinant proteins with leptospirosis serum samples. The recombinant proteins LIC11051 **(A)** and LIC11505 **(B)** were evaluated for their reactivity with leptospirosis serum sample at the onset (MAT-) and at the convalescent (MAT+) phase of the disease. Normal human serum samples (NHS) were used as control. Bound antibodies were detected by incubation with HRP-conjugated anti-human IgG (1:5000). The cutoff values, represented by the horizontal dashed line, were defined as the mean plus three standard deviations of the absorbance values of seven serum samples from NHS. Data are from three independent experiments.

To verify whether the studied proteins were expressed and immunogenic during infection, the reactivity of the recombinant proteins against leptospirosis patient sera was investigated. Paired serum samples (*n* = 16) at the onset (MAT-negative) and convalescent phase (MAT-positive) were used. rLIC11051 reacted with 37.5% of MAT-negative serum samples and 56.25% of MAT-positive serum samples (Figure 3A), while rLIC11505 showed reactivity of 50% with MAT-negative and 62.5% with MAT-positive samples (Figure 3B). The reactivity with either recombinant protein was generally consistent between paired samples. The cutoff value (dashed line) above which a sample was considered positive (0.1995 for rLIC11051 and 0.2406 for rLIC11505) was calculated based on the absorbance of seven samples from individuals with no history of leptospirosis (NHS, normal human serum). These results suggest that both proteins are expressed and are antigenic during infection.

### Evaluation of proteins in whole cell lysates and cell fraction extracts of *L. interrogans*

**Figure 4:**
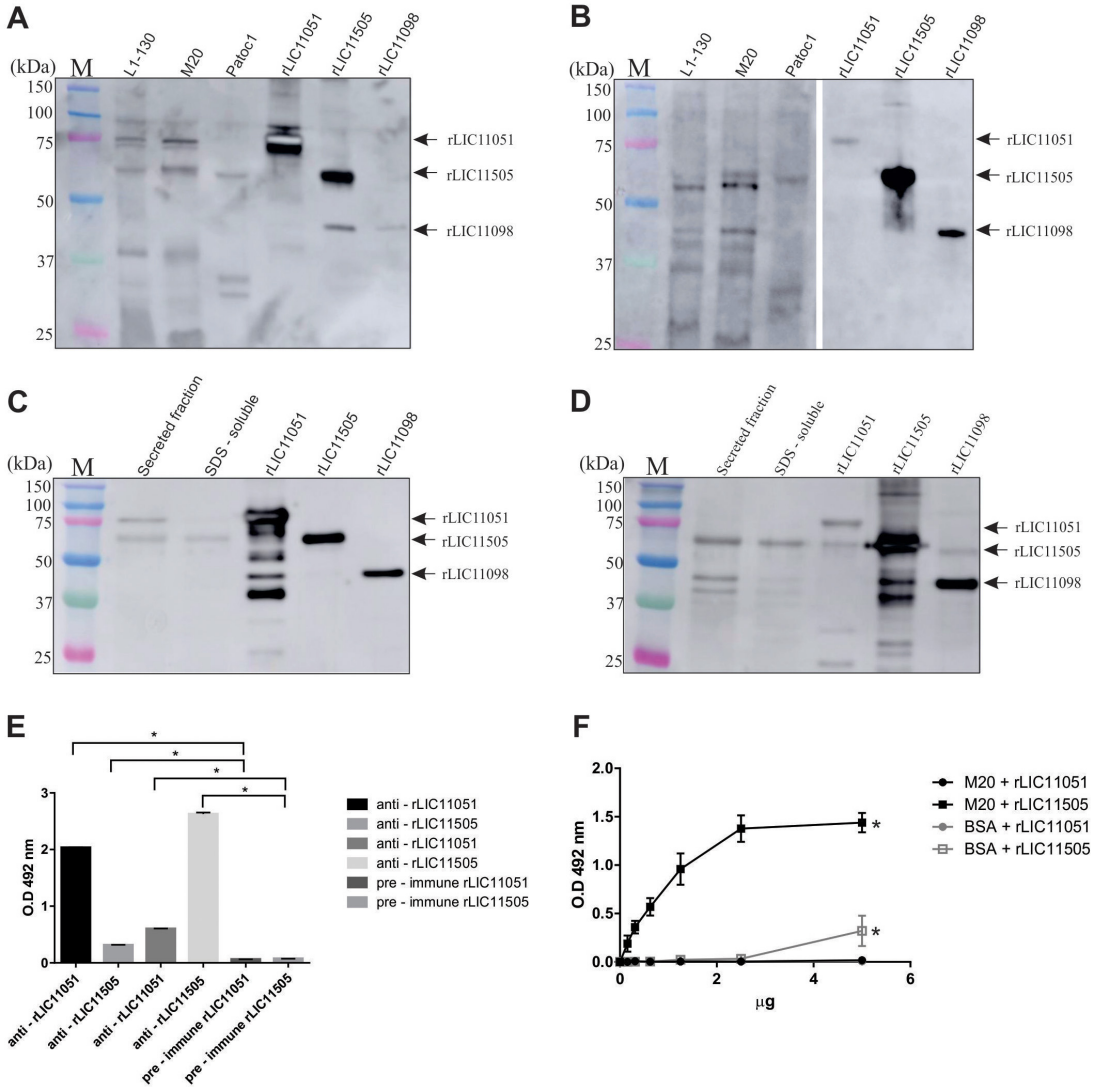
Cellular localization and cross-reactivity of polyclonal antibodies. Western blotting of whole-cell lysates of pathogenic *L. interrogans* (virulent strain L1-130 and culture-attenuated M20) and saprophytic *L. biflexa* (strain Patoc1), probed with polyclonal antibodies against **(A)** rLIC11051 and **(B)** rLIC11505, respectively. The recombinant proteins rLIC11051, rLIC11505, and rLIC11098 were used as control. Reactivity was detected by incubation with HRP-conjugated anti-mouse IgG. For subcellular location of the native proteins, western blotting was performed with secreted leptospiral protein fractions and SDS-soluble membrane proteins probed with polyclonal antibodies raised against **(C)** rLIC11051 and **(D)** rLIC11505, respectively. The purified recombinant proteins were also included. **(E)** Antibody cross-reactivity between the 2 LRR-proteins was also demonstrated by ELISA, in which 1 μg of coated recombinant protein, LIC11051 or LIC11505, were incubated with either polyclonal antisera anti-rLIC11051, -rLIC11505 or pre-immune serum, as negative control. Cross-reactivity was compared with pre-immune serum by two-tailed *t*-test; (*) representing significance (*p* < 0.05). **(F)** ELISA for binding of recombinant proteins to intact *Leptospira* cells. The binding was compared with negative controls (BSA) by two-tailed *t*-test; (*) representing significance (*p* < 0.05) only for rLIC11505.

Mouse polyclonal antiserum raised against each recombinant protein was used to detect native proteins in leptospiral whole cell lysates, including *L. interrogans* strain Fiocruz L1-130 (low-passage virulent), *L. interrogans* strain M20 (high passage culture attenuated), and saprophytic *L. biflexa*. As seen in Figure 4A, incubation with anti-rLIC11051 resulted in a band most likely corresponding to native LIC11051 in whole cell lysate from both strains of *L. interrogans*, and was absent in *L. biflexa*. For LIC11505 (Figure 4B), a band of expected molecular mass was detected in the whole-cell lysate *L. interrogans* and, at a lower intensity, in *L. biflexa*. As previously demonstrated for LRR-proteins in *Leptospira (*Eshghi et al., 2019), polyclonal antibodies cross-react with other LRR-containing proteins. It was observed that polyclonal antibodies raised against rLIC11051 could not only recognize the homologous protein but also rLIC11505 and another LRR-containing protein, rLIC11098 (Gaspar et al., 2024; Figure 4A), whereas anti-rLIC10505 antibodies reacted with rLIC11051 and rLIC11098 (Figure 4B). Additional bands were recognized in whole-cell lysates, most likely due to the conservation of LRR domains in other leptospiral proteins.

The presence of the studied proteins was also determined using virulent *L. interrogans* total secreted and membrane SDS-soluble protein samples. These cell fraction extracts were validated by immunoblotting with anti-LipL32, anti-LipL41 (outer membrane proteins), and anti-LipL31 (inner membrane protein; Supplementary Figure 1). Incubation with polyclonal antiserum anti-rLIC11051 resulted in a detectable band of ~75 kDa in the secreted protein sample, followed by faint reactivity in the SDS-soluble fraction (Figure 4C). It is worth mentioning that a band of ~60 kDa, most likely referring to LIC11505, was also observed because of the expected cross-reactivity among LRR-proteins. The purified recombinant proteins were included in the experiments (Figure 4C). The lack of predicted signal peptide for LIC11051, combined with its extracellular location, indicate that this protein could be a non-classically secreted protein (Bendtsen et al., 2005; Wang et al., 2022), which has been previously described for *Mycobacterium tuberculosis* (Harth and Horwitz, 1997), *Listeria monocytogenes* (Lenz et al., 2003), and *E. coli* (Wai et al., 2003). Upon incubation with mouse anti-rLIC11505 (Figure 4D), reactive bands compatible with the expected molecular mass were detected in both secreted and SDS-soluble fractions. The cross-reactivity between rLIC11051 and rLIC11505 was corroborated by ELISA (Figure 4E). It was also demonstrated that recombinant LIC11505 could specifically bind to intact *L. interrogans* cells in a dose-dependent manner, suggesting that the secreted native protein could reassociate to *Leptospira* surface (Figure 4F), a feature previously hypothesized for the LRR protein LIC10831(Eshghi et al., 2019). As a control, cross-reactivity of anti-LIC11051 and anti-LIC11505 sera with the His-tagged LipL41 and other non-His tagged LipL41 recombinant protein was excluded as shown in Supplementary Figure 2. Collectively, these results suggest that LIC11051 is secreted and LIC11505 were found in both fractions secreted and SDS-soluble fraction. As LIC11505 was able to reassociate to *Leptospira* surface, most probably it is an outer membrane protein, which corroborate with its *in silico* prediction. Thus, both LIC11051 and LIC11505 could play a role in pathogenesis by directly interacting with host components.

### Interaction of recombinant proteins with host components

**Figure 5:**
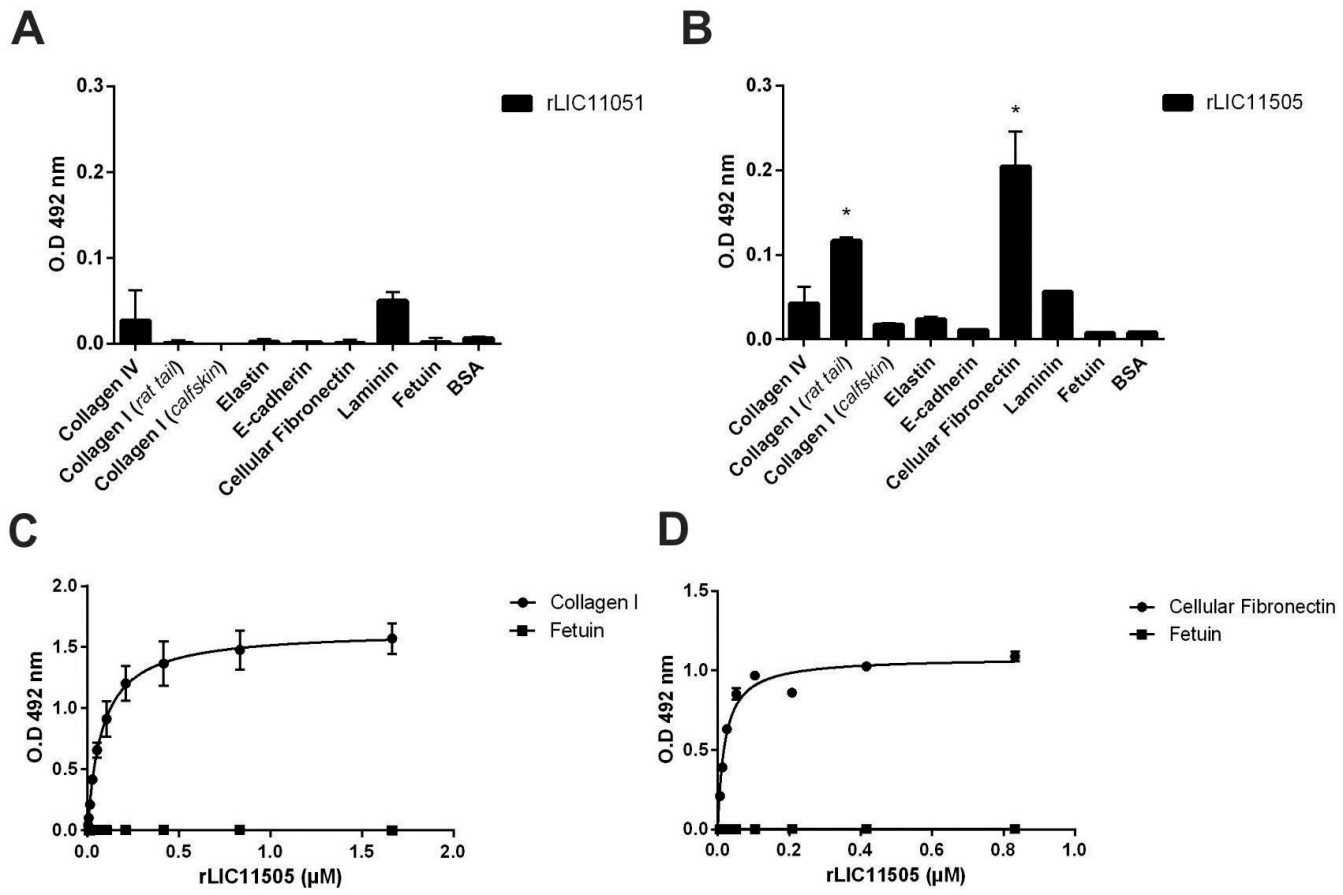
Binding of recombinant proteins to ECM and dose-dependent response evaluation. Interaction of recombinant proteins rLIC11051 **(A)** and rLIC11505 **(B)** with collagen IV, type I collagen (rat tail and calf skin), elastin, e-cadherin, cellular fibronectin and laminin, immobilized in 96-well ELISA plates. Antiserum against each recombinant protein was used to detected the interaction. Binding of recombinant proteins was compared with negative controls, fetuin and BSA, by two-tailed *t*-test; (*represents significance with fetuin, *p* < 0.05). **(C)** Dose-dependent response of rLIC11505 with collagen I (rat tail) and **(D)** with cellular fibronectin. Dose-dependent curves were fitted using GraphPad Prism software. Bars and dots represent the mean absorbance at 492 nm ± SD of three replicates. Data are from three independent experiments.

The interaction of pathogens with plasma components, the ECM, GAGs, and cell receptors is associated with host invasion, pathogen immune evasion, and colonization. These interactions are primarily mediated by secreted or surface proteins (MB et al., 2021; Vieira et al., 2009). Therefore, the ability of recombinant LIC11051 and LIC11505 to mediate their interactions with a broad range of host components was assayed. As shown in Figure 5A, rLIC11051 did not show significant binding to ECM proteins, whereas rLIC11505 (Figure 5B) showed significant binding to collagen I (rat tail) and cellular fibronectin. Binding was quantitatively confirmed by incubation with increasing concentrations of rLIC11505, with saturable and dose-dependent interactions for collagen I (Figure 5C) and cellular fibronectin (Figure 5D). The dissociation constants (K_*D*_) calculated in this study are listed in Table 1.

**Table 1:** Dissociation constants (K_*D*_) of the recombinant proteins binding to extracellular matrix, plasma, GAG, and integrin components (nM).

**Host localization**	**Component**	**rLIC11051**	**rLIC11505**
Extracellular matrix	Collagen I (rat tail)	-	79.98 ± 8.19
	Cellular fibronectin	-	20.05 ± 2.60
Plasma	Plasminogen	42.61 ± 2.72	24.25 ± 4.52
	Plasma fibronectin	-	51.09 ± 4.04
	Vitronectin	-	64.76 ± 4.65
GAG	Chondroitin sulfate	-	91.01 ± 13.37
	Chondroitin sulfate B	-	94.83 ± 12.38
	Chondroitin 4 sulfate	-	120.8 ± 9.68
	Heparin	-	12.79 ± 1.38
	Heparan sulfate	-	106.2 ± 11.99
Integrin	α8	273.5 ± 39.50	190.8 ± 9.65
	α5β1	-	73.42 ± 4.47
	αVβ1	-	133.6 ± 12.21
	αVβ3	-	151.8 ± 9.71
	αVβ5	-	108.9 ± 14.16
	αVβ6	-	74.87 ± 6.69

**Figure 6:**
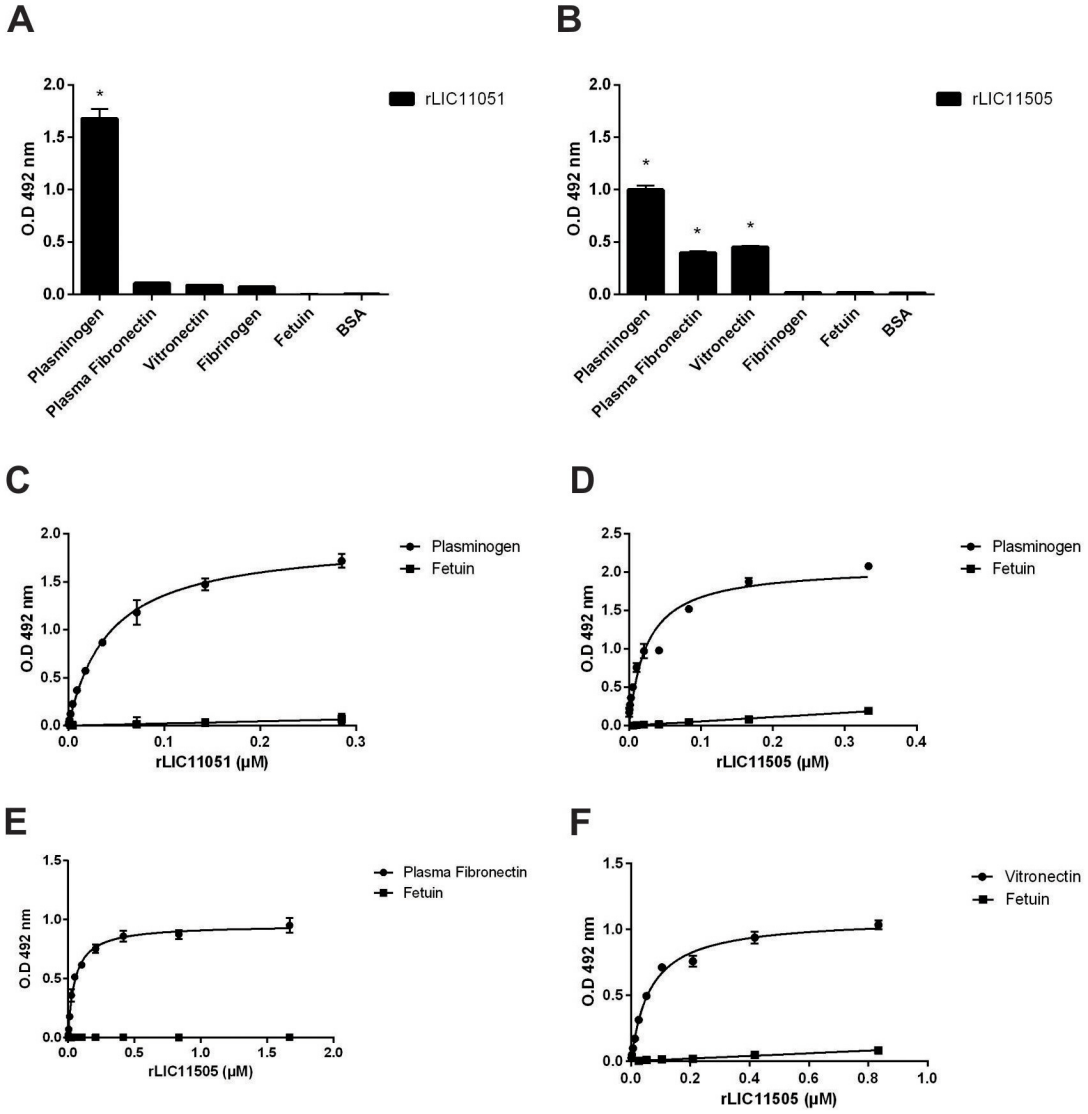
Binding of recombinant proteins to the plasma components. Interaction of recombinant proteins rLIC11051 **(A)** and rLIC11505 **(B)** with plasma components immobilized on ELISA plates. Antiserum against each recombinant protein was used to detected the interaction. Binding of recombinant proteins was compared with negative controls, fetuin and BSA, by two-tailed *t*-test; (*ast*represents significance with fetuin, *p* < 0.05). **(C)** Dose-dependent response of rLIC11051 and **(D)** rLIC11505 to PLG. **(E)** Dose-dependent response of rLIC11505 to plasma fibronectin and **(F)** to vitronectin. Dose-dependent curves were fitted using GraphPad Prism software. Bars and dots represent the mean absorbance at 492 nm ± SD of three replicates. Data from three independent experiments.

Regarding plasma components, rLIC11051 only interacted with PLG (Figure 6A), whereas rLIC11505 (Figure 6B) interacted with PLG, plasma fibronectin, and vitronectin. All binding was confirmed by dose-dependent interactions and showed saturation for PLG with rLIC11051 and rLIC11505 (Figures 6C, D, respectively), plasma fibronectin (Figure 6E), and vitronectin (Figure 6F). No significant interaction with components and regulators of the complement system, including the H factor, C3b, C4b, C4bp, C7, C8, and C9, was observed (data not shown).

### Characterization of recombinant proteins interaction with PLG

**Figure 7:**
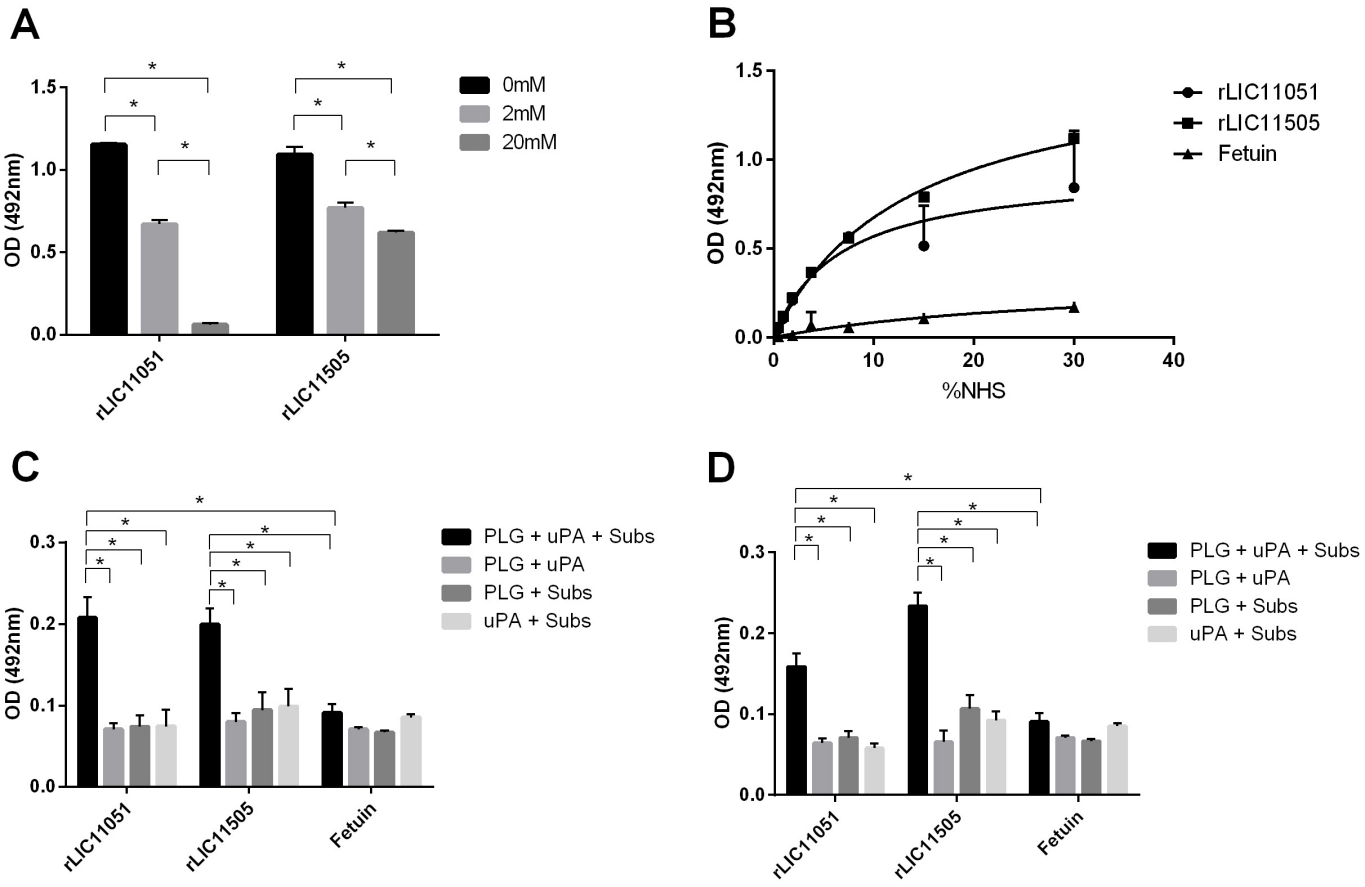
Characterization of recombinant protein interactions with PLG. **(A)** the effect of aminocaproic acid on the binding of recombinant proteins to PLG. Binding of recombinant proteins to PLG was compared to treatment without aminocaproic acid by two-tailed *t*-test (*p < 0.05). **(B)** PLG acquired from increasing concentrations of normal human serum (NHS; 0–30%) by recombinant proteins. Antiserum against each recombinant protein was used to detected the interaction. Binding of recombinant proteins to PLG was compared to fetuin by two-tailed *t*-test (*p* < 0.05). **(C)** Enzymatic assay of conversion of purified PLG bound to recombinant proteins into PLA, in the presence of uPA and PLA chromogenic substrate (D-val-leu-lys-4-nitroanilide); controls omitting one reaction component (PLG, uPA, or substrate). **(D)** Enzymatic assay of conversion of recombinant proteins bound to PLG, captured from NHS, into PLA in the presence of uPA and PLA chromogenic substrate (D-val-leu-lys-4-nitroanilide); controls omitting one reaction component (PLG, uPA, or substrate). The conversion of PLG bound to recombinant proteins into PLA was compared to fetuin/PLG by two-tailed *t*-test (*p* < 0.05). Bars represent mean absorbance at 405 nm ± SD of three replicates relative to substrate degradation. Data from three independent experiments.

Pathogenic leptospires can bind to human PLG, a zymogen that is converted into PLA on the bacterial surface in the presence of a PLG activator. Furthermore, it is well-known that PLG kringle domains mediate interactions with lysine residues of bacterial receptors (Kaarina Lahteenmaki, 2001), which has been experimentally demonstrated in *L. interrogans* (Vieira et al., 2009; Teixeira et al., 2015). As both recombinant proteins bound to PLG, their interactions were further characterized. Co-incubation with increasing concentrations of the lysine analog ACA suggested the participation of PLG kringle domains in the binding of both proteins, but only for rLIC11051 that interaction was almost completely abolished when 20 mM of ACA was used (Figure 7A). It was also observed that both recombinant proteins could capture PLG from normal human serum (NHS; Figure 7B), which more closely reflects the *in vivo* environment in a dose-dependent fashion.

To assess whether PLG bound to the studied proteins could be activated by PLA in the presence of the PLG-activator uPA, a microplate was coated with each recombinant protein or negative control fetuin and incubated with purified PLG (Figure 7C) or NHS (Figure 7D). PLA formation was indirectly evaluated by measuring the cleavage of the PLA-specific chromogenic substrate, which showed that either purified or serum PLG, when bound to both recombinant proteins, could be converted to PLA in the presence of an activator (Figures 7C, D). As expected, no significant reactivity was observed for the negative control fetuin or control reactions lacking one reaction component.

### Interaction of recombinant proteins with GAGs

**Figure 8:**
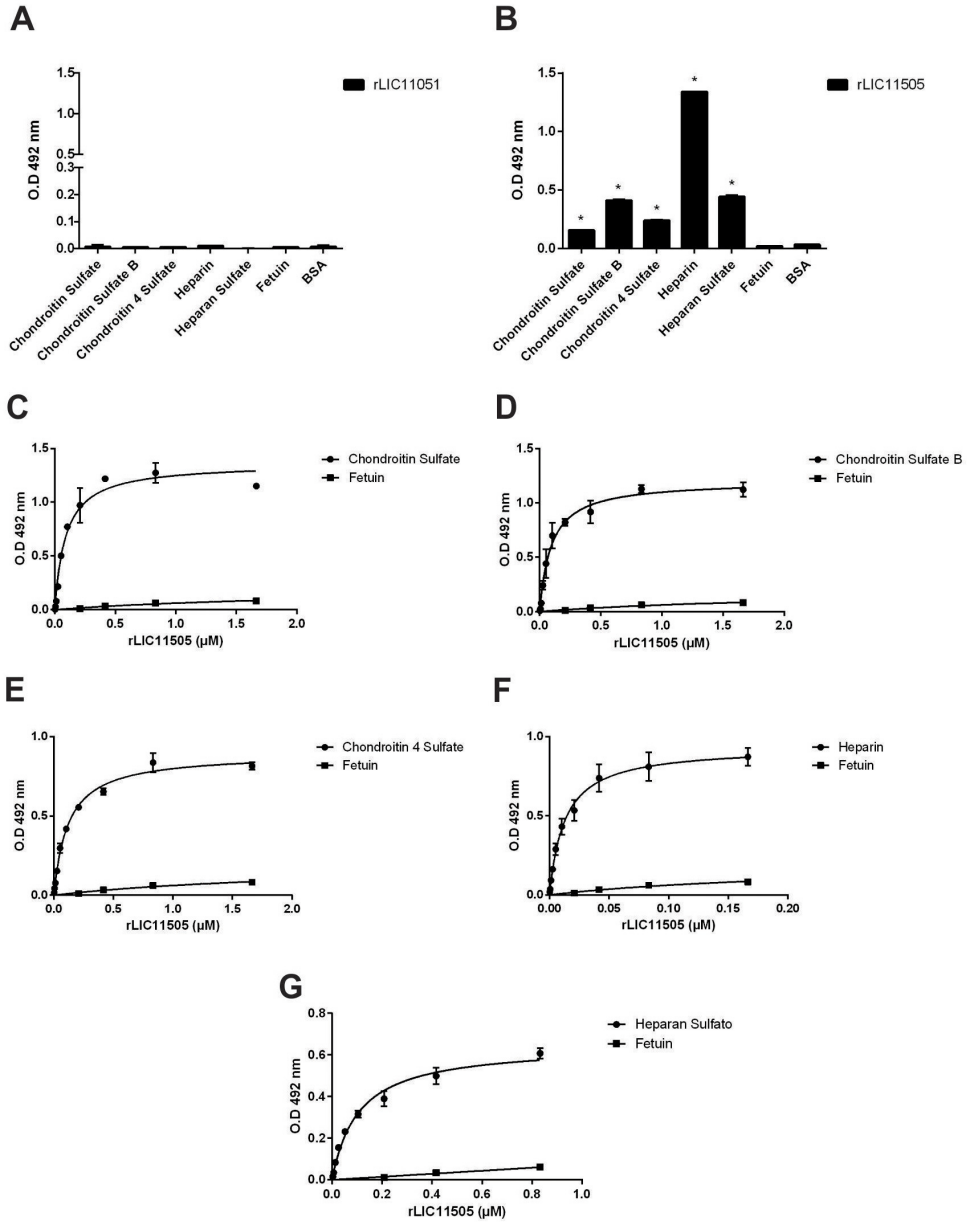
Binding of recombinant proteins to GAGs. Interaction of recombinant proteins rLIC11051 **(A)** and for rLIC11505 **(B)** with GAGs immobilized on 96-well ELISA plates. Binding of recombinant proteins was compared with the negative controls, fetuin and BSA, by two-tailed *t*-test (* represents significance with fetuin, *p* < 0.05). Dose-dependent binding of rLIC11505 with chondroitin sulfate **(C)**, chondroitin sulfate B **(D)**, chondroitin 4 sulfate **(E)**, heparin **(F)**, and heparan sulfate **(G)**. Dose dependent interactions for rLIC11505 were adjusted using GraphPad Prism software. Bars and dots represent the mean absorbance at 492 nm ± SD of three replicates. Data from three independent experiments.

GAGs are complex carbohydrates that are present on the cell surface and in the extracellular matrix, and interact with a wide range of proteins involved in physiological and pathological processes (Gandhi and Mancera, 2008). Therefore, the interaction between the recombinant proteins and GAGs was assayed. As observed in Figures 8A, B, only rLIC11505 displayed significant binding to all GAGs tested. Dose-dependent and saturable interactions were observed with chondroitin sulfate (Figure 8C), chondroitin sulfate B (Figure 8D), chondroitin 4 sulfate (Figure 8E), heparin (Figure 8F), and heparan sulfate (Figure 8G).

### Interaction of recombinant proteins with integrins

**Figure 9:**
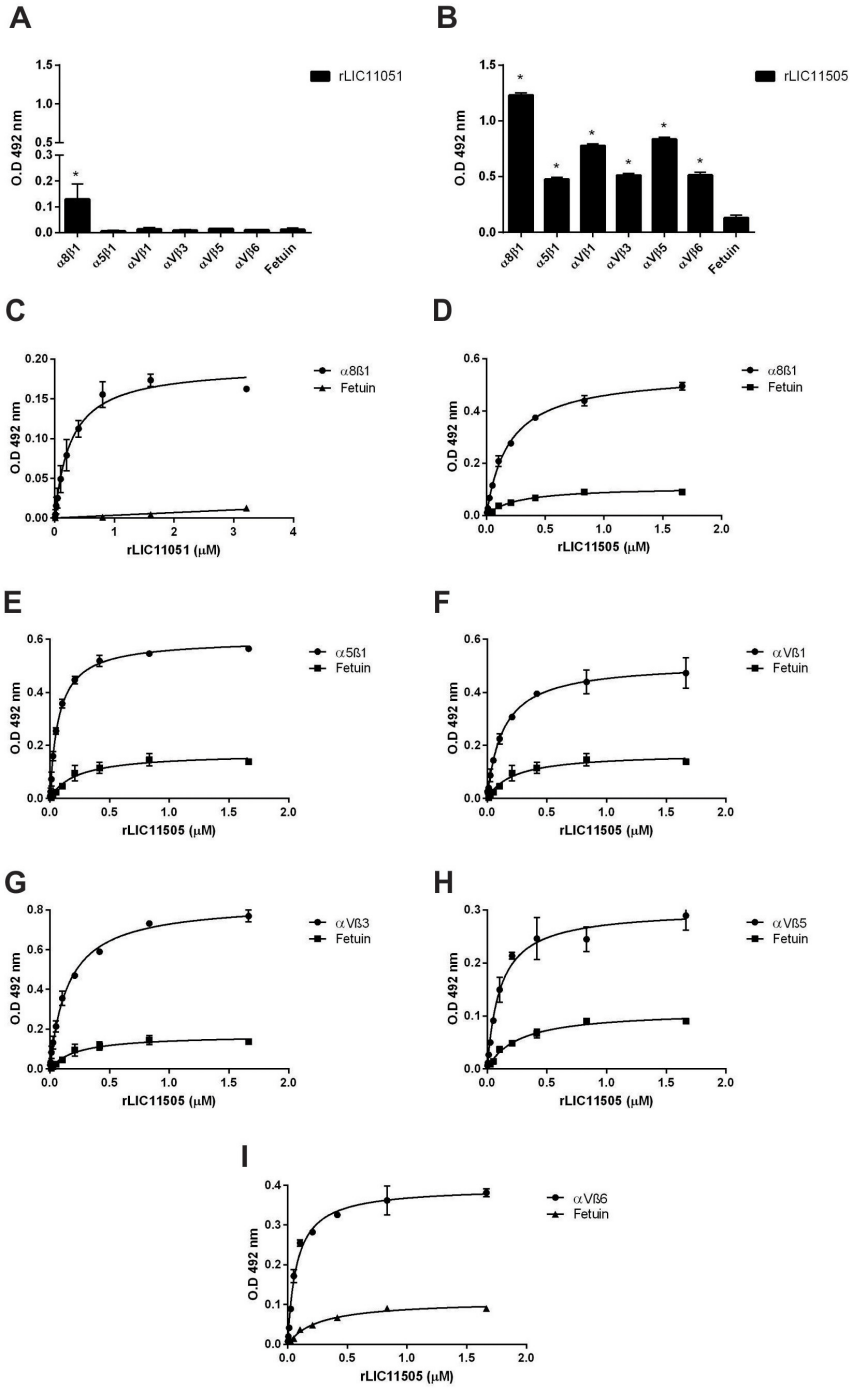
Binding of recombinant proteins to integrins. Interaction of recombinant proteins rLIC11051 **(A)** and for rLIC11505 **(B)** with α8β1, α5β1, αVβ1, αVβ3, αVβ5, and αVβ6 immobilized in 96-well ELISA plates. Binding of recombinant proteins was compared with negative control, fetuin, by two-tailed *t*-test (* represents significance with fetuin, *p* < 0.05). Dose-dependent binding of rLIC11051 **(C)** and rLIC11505 **(D)** with α8β1, and of rLIC11505 with α5β **(E)**, αVβ1 **(F)**, αVβ3 **(G)**, αVβ5 **(H)**, and αVβ6 **(I)**. The dose-dependent curves were fitted using GraphPad Prism software. Bars and dots represent the mean absorbance at 492 nm ± SD of three replicates. Data are from three independent experiments.

Integrins are cell adhesion receptors that play important roles during developmental and pathological processes and mediate the attachment of cells to the extracellular matrix (ECM). The interaction of pathogenic *Leptospira* species with this class of molecules has been previously demonstrated (Barczyk et al., 2010). Recombinant LIC11051 showed a statistically significant interaction only with α8β1, whereas rLIC11505 showed a broad-spectrum interaction with all components, as shown in Figures 9A, B. Dose-dependent binding confirmed the interaction with these components, reaching saturation for rLIC11051 (Figure 9C) and rLIC11505 (Figure 9D) with α8β1, and for rLIC11505 with α5β (Figure 9E), αVβ1 (Figure 9F), αVβ3 (Figure 9G), αVβ5 (Figure 9H), and αVβ6 (Figure 9I).

## Discussion

The LRR domain is widely distributed in bacteria, archaea, fungi, viruses, and mammals (Helft et al., 2011). Despite their shared structures, LRR-proteins can also have diverse biochemical and biological functions. One of the best-studied functions of LRR-proteins is that of immune receptors (e.g., Toll-like and NOD-like receptors) in mammals (Ng and Xavier, 2011) and plants (Zipfel and Felix, 2005).

The predominance of LRR-containing proteins in pathogenic *L. interrogans* in comparison to saprophyte *L. biflexa*, together with the variable numbers of these domains within distinct proteins, suggests their diverse and multifaceted roles in pathogenesis. Accordingly, bacterial LRR-proteins are often involved in host-pathogen interactions, displaying a multitude of ligands (Bierne et al., 2007), which also suggests that the sequences diverged during evolution to modify the protein substrates (Picardeau, 2017).

In this study, two recombinant LRR-containing proteins, LIC11051 and LIC11505, were expressed as soluble proteins and successfully purified. Both proteins were immunogenic and elicited high antibody titers after mice immunization. This immunogenicity was corroborated by the detection of human antibodies reactive to both proteins in serum samples from patients at the onset and convalescent phases of the disease, which also strongly suggests that LIC11051 and LIC11505 are expressed during infection. As observed by Miras et al. (2015) and Eshghi et al. (2019) with other LRR-proteins, cross-reactivity between the two proteins was observed, most likely due to the presence of the LRR domains. Thus, it is worth mentioning that the recognition of the studied proteins by human antibodies could be due to homologous antibodies as well as antibodies against other expressed LRR-proteins during infection.

The native proteins LIC11051 and LIC11505, which are conserved among pathogenic strains, were detected in the total cell lysate of either low- or high-passage *L. interrogans*. In addition, both proteins were characterized as exoproteins and were detected in the secreted protein fractions of virulent *L. interrogans*. Interestingly, the recombinant protein LIC10505 displayed strong binding to intact leptospiral cells, suggesting that native secreted proteins may be reassociated to leptospires during infection. This feature was previously demonstrated for enolases from *Leptospira* (Nogueira et al., 2013) and *Streptococcus pneumoniae* (Kornblatt et al., 2011). The bacterial surface receptors secreted by LIC10505 remain to be determined.

Interactions between pathogens and host components are crucial for adhesion, invasion, and immune evasion (Chagnot et al., 2012). Many pathogens express proteins that interact with the ECM, which provides structural support for cell adhesion, migration, and signaling and acts as a protective barrier (Patti et al., 1994; Singh et al., 2012). Our study showed that the rLIC11505 protein binds significantly to ECM components such as collagen type I, fibronectin, and GAGs (Chagnot et al., 2012), which may be associated with pathogenicity, as observed in other organisms (Tomlin and Piccinini, 2018). Fibronectin is a common target for microorganisms, and this interaction has already been demonstrated for some leptospiral proteins such as Lsa66, LigA, LigB, LipL21, and LipL41 (Choy et al., 2007; Oliveira et al., 2011; MB et al., 2021), which are considered important for leptospiral virulence (Fernandes et al., 2022, 2023). Moreover, bacterial proteins bind to collagen type I to anchor and invade tissues (Antonara et al., 2011). In addition to rLIC11505, several other *Leptospira* recombinant proteins such as OmpL1, Lsa21, and LigA displayed binding to multiple collagen types (Choy et al., 2007; Atzingen et al., 2008; Fernandes et al., 2012; Daroz et al., 2021).

Proteoglycans are ubiquitous among animal cells, and as discussed below, their carbohydrate chains, GAGs, are frequent targets for binding by bacteria (Rostand and Esko, 1997; Fagan et al., 2008). Several pathogens express surface proteins that recognize GAGs, including *Neisseria gonorrhoeae* (Van Putten and Paul, 1995), *Bordetella pertussis, Mycobacteria* spp. (Menozzi et al., 2002), *Listeria monocytogenes* (Alvarez-Dominguez et al., 1997), and enterotoxigenic *E. coli* (Fleckenstein et al., 2002). The spirochete *Borrelia burgdorferi* expresses the GAG-binding adhesin BBK32, which is involved in joint colonization (Lin et al., 2015). The interaction of pathogenic leptospires with GAGs has been previously reported, and OmpL1was identified as a GAG-binding protein (Breiner et al., 2009; Robbins et al., 2015). Similar to LipL21, rLIC11505 interacted with several GAG components (MB et al., 2021).

When leptospires come into contact with the host, they encounter barriers, such as the skin, mucous membranes, and blood vessels. After overcoming these barriers, they enter the bloodstream where they interact with plasma molecules and components of the complement system involved in coagulation and fibrinolysis (Schaller et al., 2008). The recombinant proteins studied interacted with PLG, a key host molecule in the fibrinolytic system employed by microorganisms for invasion and establishment of infection. Spirochetes such as *Borrelia spp*. and *Treponema denticola* bind to PLG to increase their infectivity (Coleman et al., 1995; Klempner et al., 1996; Fenno et al., 2000). Our group was the first to describe the ability of *Leptospira* spp. to acquire PLG from human plasma (Vieira et al., 2009). Leptospira-bound PLG can be converted to PLA, which decomposes fibrin clots and ECM components, helping evade the immune response (Ponting, 1992; Vieira et al., 2011, 2013). Thus, the ability of recombinant proteins to interact with PLG represents a crucial step in invasion and establishment of infection. Moreover, other proteins found in the ECM, such as vitronectin, regulate the complement pathway by inhibiting deposition of the attack complex in the ECM (Milis et al., 1993).

Integrins are a large family of proteins that play important roles in several biological processes by binding various ligands (Barczyk et al., 2010). Many of these interactions occur through the RGD motif present in the ECM components and are crucial for cellular communication. The recombinant LIC12254 protein from *L. interrogans* binds to αVβ8 and α8β1 human integrins via the RGD motif. When activated, integrins trigger intracellular signaling that causes rearrangement of the cytoskeleton to facilitate internalization by microorganisms (Geiger et al., 2009). Accordingly, rLIC11505, a multireceptor-binding protein that interacts with all tested integrins, may be involved in this process.

In conclusion, although the LIC11051 protein displayed a limited ligand range, its presence restricted to subclade P1 suggests that the protein is important for virulence. The interaction of LIC11505 with multiple host components, which might be correlated with its higher number of LRR domains, highlights the complexity of the infection and immune evasion strategies employed by pathogenic *Leptospira*. These proteins not only aid in tissue adhesion and invasion but can also modify the host fibrinolytic system, which may contribute to infection development. Future studies using recent CRISPR techniques to generate leptospiral mutants for both proteins will shed light on their roles as virulence factors and confirm the *in vivo* participation of these proteins in the pathogenesis process.

## Data Availability

The datasets presented in this study can be found in online repositories. The names of the repository/repositories and accession number(s) can be found in the article/Supplementary material.

## Supplementary material

Supplementary Figure 1 Validation of secreted protein fractions and SDS-soluble membrane proteins by immunoblotting. In **(A)**, anti-LipL31 antibodies (1:1000; inner membrane protein); in **(B)**, anti-LipL32 (1:1000) and anti-LipL41 (1:1000; outer membrane proteins). Total cell lysate of *L. interrogans*, Fiocruz L1-130 and culture-attenuated M20 strains were used as a control.

Supplementary Figure 2 Analysis of cross-reactivity of antibodies anti-rLIC11051 and rLIC11505 against a non-LRR, a His-tagged and non-His-tagged recombinant proteins. Binding of recombinant proteins (250 ng), performed in triplicate, was incubated with anti-rLIC11051 and anti-rLIC11505 antibodies (1:20000). Proteins without predictions of LRR domains such as His-tagged LipL41, a non-His tagged LipL 41 were used. Cross-reactivity was compared with negative controls fetuin and BSA by two-tailed *t*-test; (^*^) representing significance (*p* < 0.05).
