# Supplementary figures and images for: Leucine-rich repeat proteins of Leptospira interrogans that interact to host glycosaminoglycans and integrins
Source: Front Microbiol. 2024 Nov 26;15:1497712. doi: 10.3389/fmicb.2024.1497712 (PMC11629876; doi:10.3389/fmicb.2024.1497712)

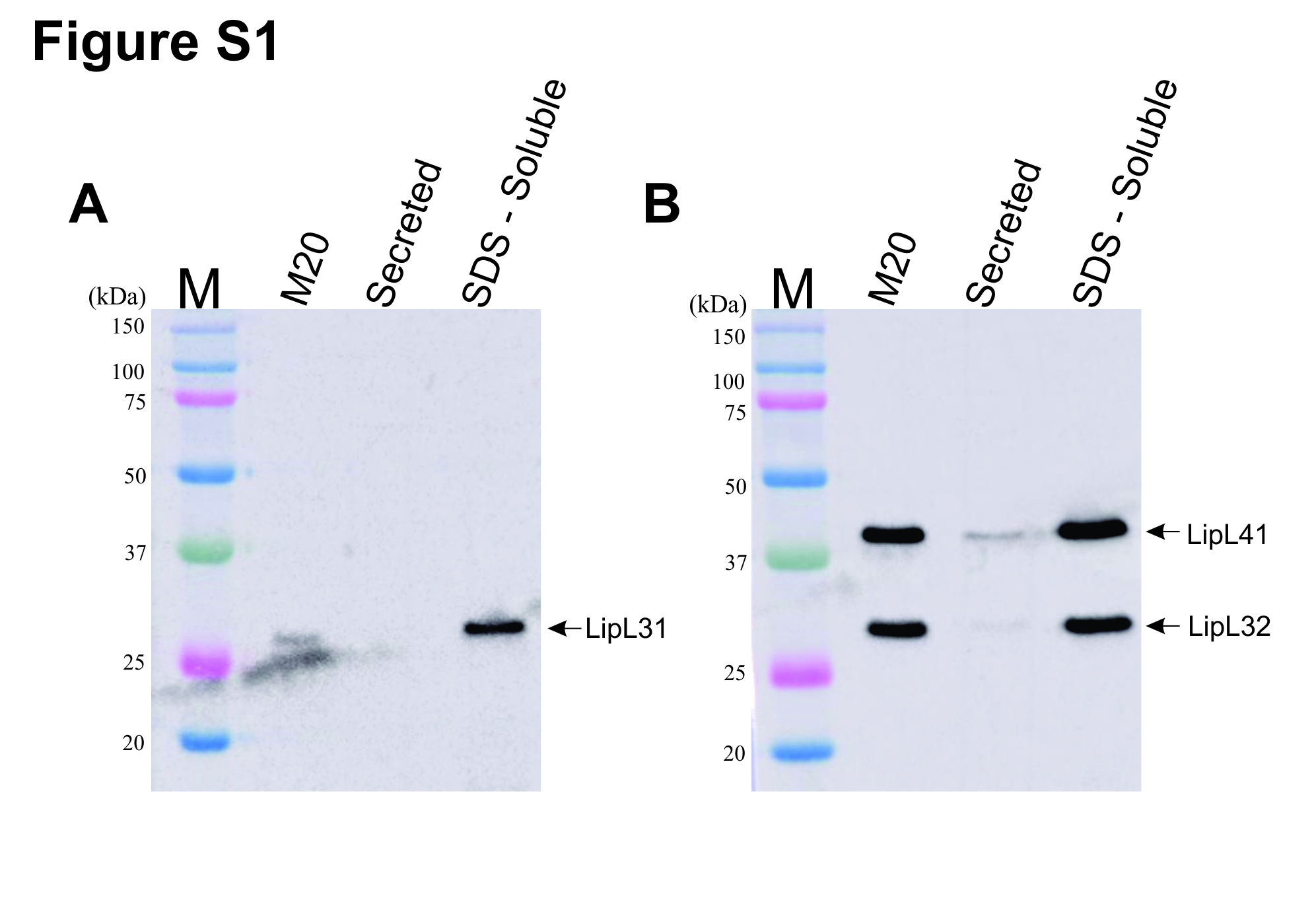

Supplement: Supplementary Figure 1 — Validation of secreted protein fractions and SDS-soluble membrane proteins by immunoblotting. In (A), anti-LipL31 antibodies (1:1000; inner membrane protein); in (B), anti-LipL32 (1:1000) and anti-LipL41 (1:1000; outer membrane proteins). Total cell lysate of L. interrogans, Fiocruz L1-130 and culture-attenuated M20 strains were used as a control. [file Image_1.jpeg]

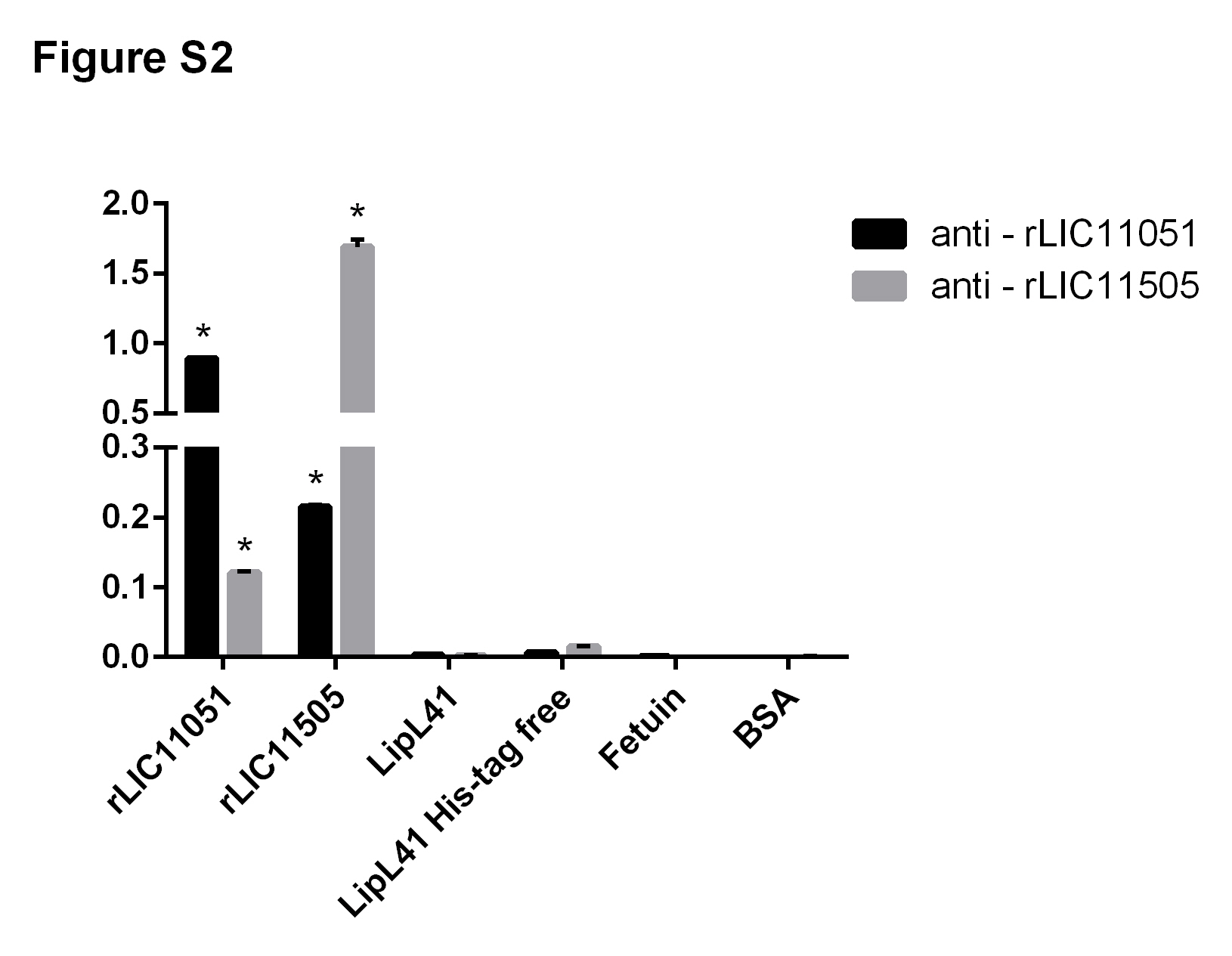

Supplement: Supplementary Figure 2 — Analysis of cross-reactivity of antibodies anti-rLIC11051 and rLIC11505 against a non-LRR, a His-tagged and non-His-tagged recombinant proteins. Binding of recombinant proteins (250 ng), performed in triplicate, was incubated with anti-rLIC11051 and anti-rLIC11505 antibodies (1:20000). Proteins without predictions of LRR domains such as His-tagged LipL41, a non-His tagged LipL 41 were used. Cross-reactivity was compared with negative controls fetuin and BSA by two-tailed t-test; (*) representing significance (p < 0.05). [file Image_2.jpeg]
